# Carboplatin Monotherapy Induced Complete Response in Elderly Metastatic Seminoma and CKD: A Case Report

**DOI:** 10.1002/iju5.70241

**Published:** 2026-08-05

**Authors:** Gento Omae, Kosuke Kitamura, Satoru Muto

**Affiliations:** ^1^ Department of Urology Juntendo University Nerima Hospital Tokyo Japan

**Keywords:** carboplatin, chronic kidney disease, elderly patients, metastatic testicular cancer, seminoma

## Abstract

**Background:**

Cisplatin‐based chemotherapy is the standard treatment for metastatic seminoma but may be unsuitable for elderly patients with comorbidities. We report a case of metastatic seminoma with chronic kidney disease (CKD) in an elderly patient successfully treated with carboplatin monotherapy.

**Case Presentation:**

A 70‐year‐old man presented with a left testicular tumor and para‐aortic lymphadenopathy, causing left‐sided hydronephrosis. Radical left high orchiectomy confirmed stage IIC pure seminoma pT1N3M0 with a favorable prognosis according to the International Germ Cell Cancer Collaborative Group classification. Because renal impairment and poor performance status precluded cisplatin‐based chemotherapy, the patient received four cycles of carboplatin monotherapy (AUC 7). Grade 2–3 pancytopenia occurred but was manageable with pegfilgrastim and dose interval adjustment. Post‐treatment PET‐CT showed no residual uptake. Consolidation para‐aortic radiotherapy was subsequently performed. The patient remains disease‐free 1.5 years after treatment.

**Conclusion:**

Carboplatin monotherapy may be a feasible alternative for metastatic seminoma patients who are unsuitable for cisplatin‐based chemotherapy.

## Keynote Message

Elderly patients with metastatic seminoma and CKD are often unsuitable for standard cisplatin‐based chemotherapy. This case demonstrates that carboplatin monotherapy achieved a durable complete response with acceptable toxicity in such high‐risk patients. Carboplatin monotherapy may represent a reasonable alternative for carefully selected patients with significant comorbidities.

## 1 Background

Testicular cancer accounts for only 1% of all male malignancies and most commonly affects younger men [1, 2]. Although the mean age at diagnosis is increasing, testicular germ cell tumors in patients older than 50 years remain uncommon, and only a small proportion occur in patients aged ≥ 70 years [3, 4]. Seminoma is the predominant histological subtype in elderly patients [3, 4]. Despite generally favorable biology, outcomes in older patients are not always superior because treatment tolerance may be reduced by aging and comorbidities [5, 6, 7].

Cisplatin‐based chemotherapy is the standard treatment for metastatic seminoma, offering high cure rates. However, cisplatin is associated with significant nephrotoxicity, neurotoxicity, and hematologic toxicity, which may limit its use in elderly patients with impaired renal function [1, 2]. Carboplatin has a more favorable toxicity profile and has been investigated as an alternative platinum agent in seminoma. We report a case of metastatic seminoma CKD successfully treated using carboplatin monotherapy.

## 2 Case Presentation

**FIGURE 1:**
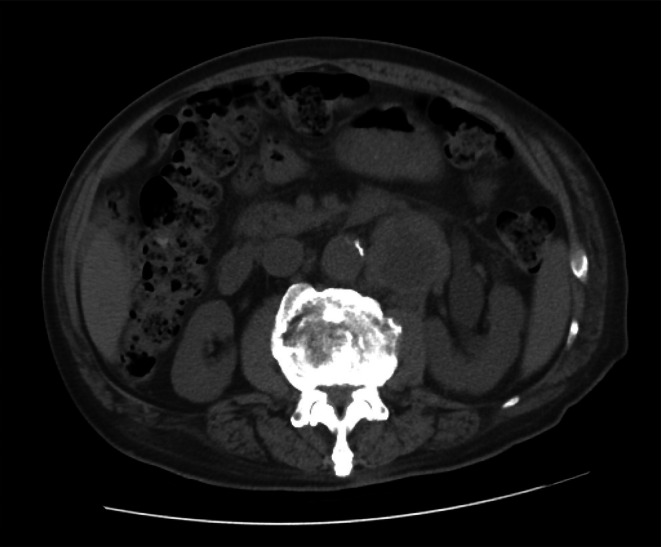
Preoperative CT‐scan image showing para‐aortic lymphadenopathy and left hydronephrosis.

A 70‐year‐old man was referred to our institution with impaired renal function and a left testicular mass. Computed tomography (CT) scan revealed a left testicular tumor with multiple metastatic lymph nodes, including para‐aortic lymphadenopathy measuring 5.5 cm, causing grade 2 left‐sided hydronephrosis. The right kidney showed no structural abnormalities, although urinalysis demonstrated proteinuria (2+), suggesting underlying CKD (Figure 1). Performance status was 2–3. Scrotal ultrasound revealed a heterogeneous left testicular mass. Among all the serum tumor markers, only human chorionic gonadotropin (hCG) was elevated (41.4 mIU/mL). Laboratory findings showed serum creatinine 2.45 mg/dL and estimated glomerular filtration rate 21.5 mL/min/1.73 m^2^. Hemoglobin was mildly decreased at 10.3 g/dL.

Radical left high orchiectomy was performed without complications. Postoperative hCG remains elevated (30.1 mU/mL) but has decreased. Histopathology confirmed pure seminoma, and imaging findings were consistent with clinical stage IIC disease (pT1N3M0). According to the International Germ Cell Cancer Collaborative Group (IGCCCG) classification, the prognosis was favorable.

Because of severe renal dysfunction and poor performance status, the patient was considered unsuitable for the standard cisplatin‐based chemotherapy. Percutaneous nephrostomy was considered but deferred due to concerns regarding long‐term management and the patient's overall condition. Carboplatin monotherapy at AUC 10 was initially planned; however, the dose was reduced to AUC 7 because of frailty and renal impairment.

**FIGURE 2:**
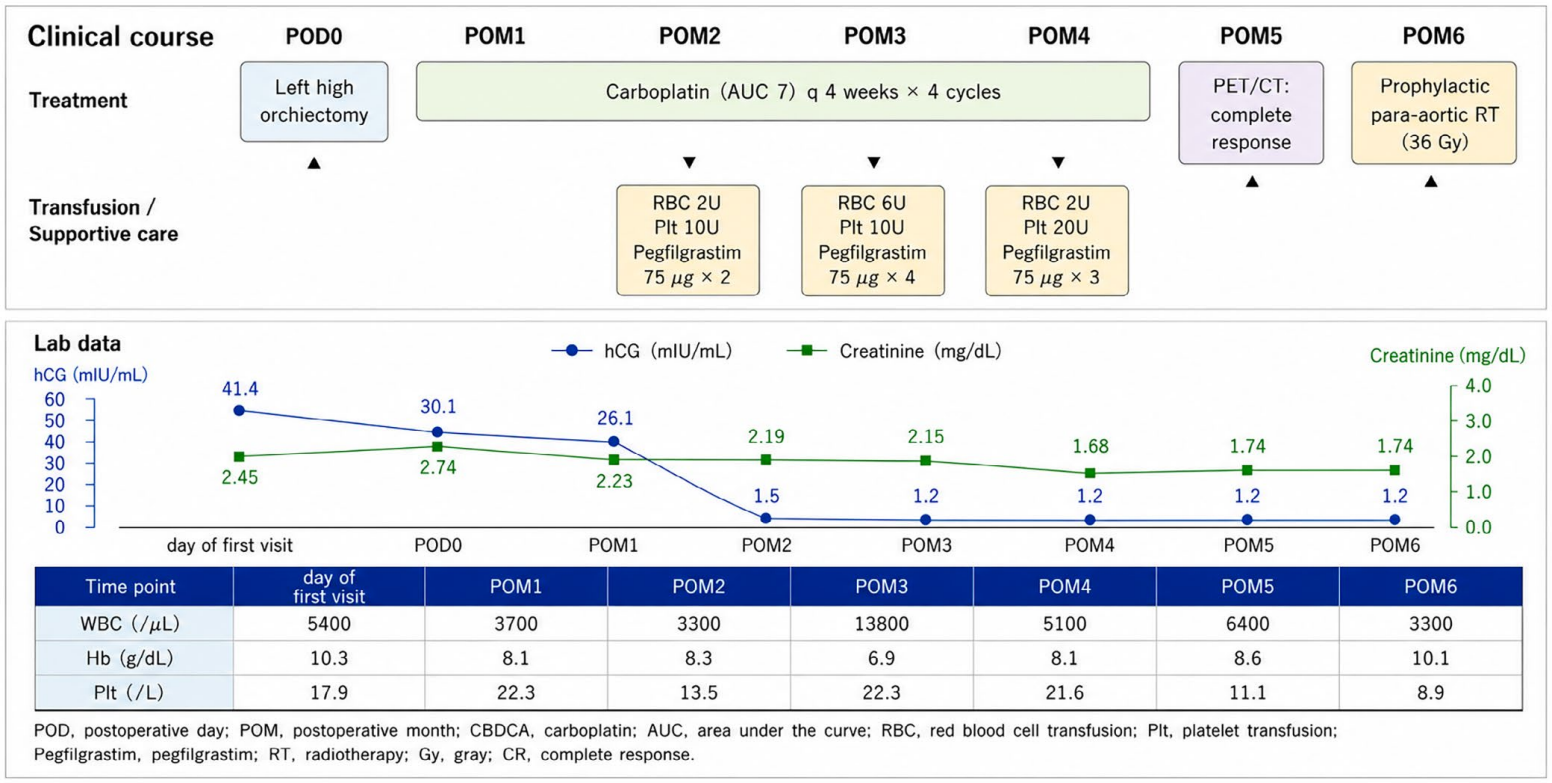
Clinical course of the patient. The figure summarizes the treatment timeline, changes in tumor markers, renal function, and major adverse events during carboplatin monotherapy.

The patient received four cycles of carboplatin monotherapy every 28 days (Figure 2). Grade 2–3 pancytopenia developed during each cycle and was managed with prophylactic pegfilgrastim and supportive care. No treatment‐related severe nonhematologic toxicities occurred. During the second cycle, the patient experienced a fall resulting in right optic nerve trauma unrelated to chemotherapy.

**FIGURE 3:**
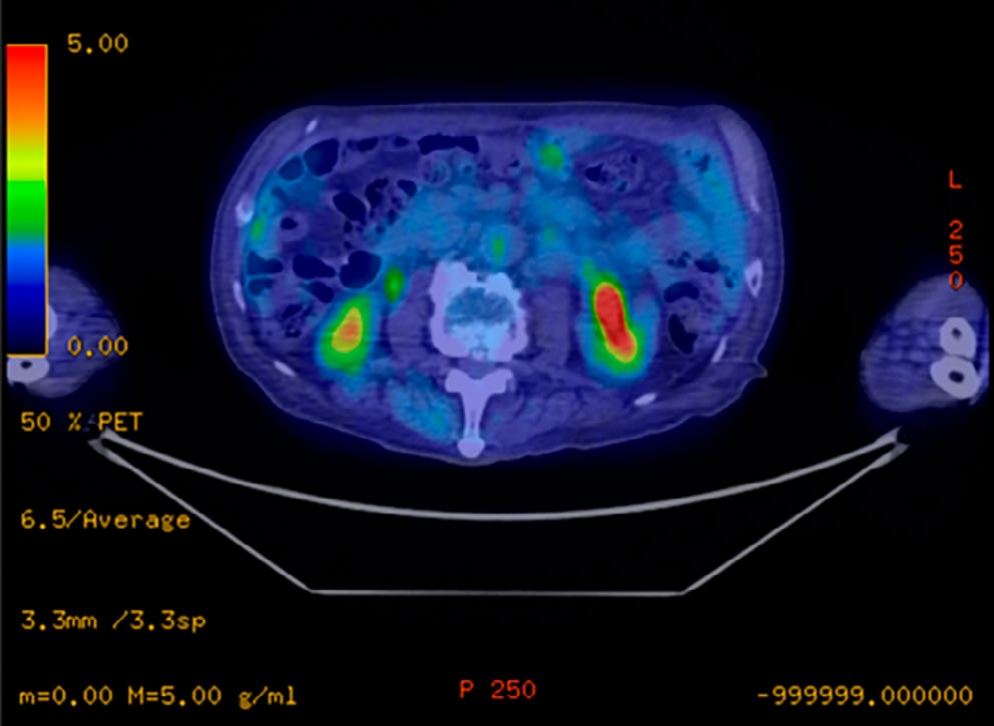
PET‐CT scan image showing no tracer accumulation in abdominal lymph nodes.

Post‐treatment Positron Emission Tomography‐CT (PET‐CT) scan showed no abnormal uptake in abdominal lymph nodes, with reduction in size to 2.8 cm. Hydronephrosis persisted but improved to grade 1 (Figure 3). Serum hCG normalized after treatment. Renal function remained impaired despite tumor response. To reduce the risk of residual microscopic disease while avoiding additional systemic toxicity, consolidation para‐aortic radiotherapy (36 Gy) was administered. The patient remains disease‐free 1.5 years after treatment.

## 3 Discussion

Testicular germ cell tumors are uncommon in elderly patients, as the peak incidence typically occurs in younger men. Previous studies demonstrated that seminoma is the predominant histological subtype among older patients with germ cell tumors [1, 2] (Table 1). Kawai et al. [3] reported that patients aged ≥ 50 years represented a relatively small proportion of all testicular germ cell tumors in a large Japanese cohort, with seminoma occurring more frequently than non‐seminoma. Similarly, Berney et al. [4] showed that seminoma was the most common histology among patients aged ≥ 60 years.

**TABLE 1:** Elderly seminoma patients reported in previous studies.

Study	Definition of elderly	No. of patients	Main histology	Major findings
Kawai et al. (2017)	≥ 50 years	1143	Seminoma predominant	Elderly patients accounted for a small proportion of testicular GCT cases, with seminoma more frequently observed than non‐seminoma
Berney et al. (2008)	≥ 60 years	50	Seminoma predominant	Seminoma was the most common histology in elderly patients, whereas non‐seminomatous tumors were relatively uncommon
Feldman et al. (2013)	≥ 50 years	50	Seminoma and non‐seminoma	Platinum‐based chemotherapy was feasible, although elderly patients experienced increased hematologic and renal toxicities
NCCN Guidelines (2020)	Not specified	N/A	Seminoma and non‐seminoma	Cisplatin‐based chemotherapy remains the standard treatment for metastatic seminoma
ESMO‐EURACAN Guidelines (2022)	Not specified	N/A	Seminoma and non‐seminoma	Carboplatin is generally considered less effective than cisplatin for metastatic seminoma but may be considered in selected patients unfit for cisplatin
Present case	70 years	1	Pure seminoma	Carboplatin monotherapy followed by consolidative radiotherapy achieved complete metabolic response despite CKD

Cisplatin‐based chemotherapy remains the standard treatment for metastatic seminoma according to current NCCN and ESMO guidelines. However, elderly patients frequently have comorbidities and reduced organ function that may limit cisplatin eligibility. Feldman et al. [5] demonstrated that platinum‐based chemotherapy in older patients was associated with increased hematologic and renal toxicities.

Carboplatin, a second‐generation platinum analog, has a more favorable toxicity profile and is widely used in other malignancies such as urothelial carcinoma. Although carboplatin‐based regimens are considered inferior in non‐seminomatous germ cell tumors, several studies have evaluated its efficacy in seminoma. Horwich et al. [6] demonstrated inferior three‐year survival in patients with favorable‐risk non‐seminomatous GCTs treated with CEB (carboplatin (AUC 5), etoposide, bleomycin) compared to BEP. Bokemeyer et al. [7] also reported higher rates of relapse and treatment‐related mortality with CEB (carboplatin AUC 5), though response to chemotherapy itself was similar.

In contrast, Giesen et al. [8] showed that patients with advanced seminoma treated with CEB (AUC 5) had comparable overall survival (OS) and progression‐free survival (PFS) to cisplatin‐based regimens, with reduced toxicity. Bokemeyer et al. [9] evaluated carboplatin monotherapy (AUC 7 or 400 mg/m^2^) in advanced seminoma, which yielded inferior five‐year OS and PFS. However, a recent 2022 study by Alifrangis et al. [10] involving 216 patients reported that carboplatin monotherapy at AUC 10 achieved similar efficacy to cisplatin‐based treatment with fewer complications. Although perspective randomized controlled trials are needed to establish this as standard treatment, it suggests the effectiveness of carboplatin‐based treatment for advanced seminoma.

In most of the studies regarding metastatic seminoma, patients over 70 years old received standard cisplatin‐based chemotherapy with careful management of toxicities. Ismaili et al. [11] reported a 78‐year‐old man with hepatic and renal dysfunction successfully managed with two cycles of carboplatin monotherapy (AUC 7), followed by two cycles of carboplatin (AUC 5) with etoposide, which also backed up the potential of carboplatin monotherapy as an alternative for elderly patients.

In our case, the patient was unsuitable for cisplatin and bleomycin due to renal dysfunction and poor performance status. Carboplatin monotherapy at AUC 10 was initially considered, but the dose was reduced to AUC 7. A total of four cycles of carboplatin monotherapy (AUC 7) resulted in effective disease control with manageable toxicity. Although dose and schedule modifications were necessary, the treatment outcome was favorable, suggesting that carboplatin monotherapy may be a viable alternative in select cases.

Cisplatin‐based chemotherapy remains the standard treatment for clinical stage IIC seminoma. However, elderly patients with significant renal impairment may not tolerate cisplatin because of its nephrotoxicity. In the present case, carboplatin monotherapy was selected as an alternative systemic therapy. Although radiotherapy is not routinely recommended for stage IIC disease, seminoma is highly radiosensitive. Therefore, consolidation radiotherapy was performed after systemic therapy to minimize the risk of residual microscopic disease while avoiding additional systemic toxicity.

## 4 Conclusion

1. Germ cell tumors are rare but can be found in elderly patients aged 70 or older.

2. Cisplatin‐based chemotherapy remains the standard treatment for metastatic seminomas; however, carboplatin monotherapy offers a promising alternative for patients with comorbidities such as CKD, ensuring effective treatment with reduced toxicities.

## Ethics Statement

The authors have nothing to report.

## Consent

Written informed consent was obtained from the patient for publication of this case report.

## Conflicts of Interest

The authors declare no conflicts of interest.

## Data Availability

Data sharing is not applicable to this article, as no datasets were generated or analyzed during the current study.
